# Antiquity of Anæsthetic Agents

**Published:** 1849-07

**Authors:** 


### ANTIQUITY OF ANÆSTHETIC AGENTS.

A recent number of the London Medical Gazette states that Mr. Stanislaus Julien makes the following statement. A Chinese Surgeon, upwards of two hundred years before our era, had employed the canabis Indici to produce a state of insensibility before performing surgical operations. This curious affair is extracted from a Chinese system of medicine, entitled, *Kou-kin-i-long*, which was published in the sixteenth century. From the representations of the delightful reveries which the patient experiences while under the influence of that drug; some of them being recent, and from highly respectable sources, we are led to hope that some one may commence a course of experimental observations upon the effects of the Indian hemp, with reference to its anæsthetic properties.

The Boston Medical and Surgical Journal states that in New England it has been found to be nearly or quite powerless, and adds that the want of success may possibly be in consequence of the timidity of practitioners inducing them to prescribe doses not of sufficient potency. From various sources abroad, the impression has become extensively diffused, that the canabis Indici exerts a powerful control over the nervous system, which might be readily confirmed or removed. Here is ground for investigation.—*Dental Recorder.*

